# Core Genome MLST for Source Attribution of *Campylobacter coli*

**DOI:** 10.3389/fmicb.2021.703890

**Published:** 2021-07-13

**Authors:** Lucas Harrison, Sampa Mukherjee, Chih-Hao Hsu, Shenia Young, Errol Strain, Qijing Zhang, Glenn E. Tillman, Cesar Morales, Jovita Haro, Shaohua Zhao

**Affiliations:** ^1^U.S. Food and Drug Administration, Center for Veterinary Medicine, Laurel, MD, United States; ^2^College of Veterinary Medicine, Iowa State University, Ames, IA, United States; ^3^U.S. Department of Agriculture, Food Safety and Inspection Service, Athens, GA, United States

**Keywords:** *Campylobacter coli*, source attribution, cgMLST, food safety, antibiotic resistance, MMD

## Abstract

*Campylobacter* species are among the leading foodborne bacterial agents of human diarrheal illness. The majority of campylobacteriosis has been attributed to *Campylobacter jejuni* (85% or more), followed by *Campylobacter coli* (5–10%). The distribution of *C. jejuni* and *C. coli* varies by host organism, indicating that the contribution to human infection may differ between isolation sources. To address the relative contribution of each source to *C. coli* infections in humans, core genome multilocus sequence type with a 200-allele difference scheme (cgMLST_200_) was used to determine cgMLST type for 3,432 *C. coli* isolated from food animals (*n* = 2,613), retail poultry meats (*n* = 389), human clinical settings (*n* = 285), and environmental sources (*n* = 145). Source attribution was determined by analyzing the core genome with a minimal multilocus distance methodology (MMD). Using MMD, a higher proportion of the clinical *C. coli* population was attributed to poultry (49.6%) and environmental (20.9%) sources than from cattle (9.8%) and swine (3.2%). Within the population of *C. coli* clinical isolates, 70% of the isolates that were attributed to non-cecal retail poultry, dairy cattle, beef cattle and environmental waters came from two cgMLST_200_ groups from each source. The most common antibiotic resistance genes among all *C. coli* were *tetO* (65.6%), *bla*_*OXA*__–__193_ (54.2%), *aph(3′)-IIIa* (23.5%), and *aadE-Cc* (20.1%). Of the antibiotic resistance determinants, only one gene was isolated from a single source: *bla*_*OXA*__–__61_ was only isolated from retail poultry. Within cgMLST_200_ groups, 17/17 cgMLST_200_-435 and 89/92 cgMLST_200_-707 isolates encoded for *aph(3’)-VIIa* and 16/16 cgMLST_200_-319 harbored *aph(2’)-If* genes. Distribution of *bla*_*OXA*_ alleles showed 49/50 cgMLST_200_-5 isolates contained *bla*_*OXA*__–__498_ while *bla*_*OXA*__–__460_ was present in 37/38 cgMLST_200_-650 isolates. The cgMLST_200_-514 group revealed both *ant*(6)*-Ia* and *sat4* resistance genes in 23/23 and 22/23 isolates, respectively. Also, cgMLST_200_-266 and cgMLST_200_-84 had GyrAT86I mutation with 16/16 (100%) and 14/15 (93.3%), respectively. These findings illustrate how cgMLST and MMD methods can be used to evaluate the relative contribution of known sources of *C. coli* to the human burden of campylobacteriosis and how cgMLST typing can be used as an indicator of antimicrobial resistance in *C. coli*.

## Introduction

*Campylobacter* species are spiral or rod-shaped gram-negative bacteria with optimal growth conditions in microaerophilic environments at 30–47°C (Hsieh et al., 2018). Despite these growth requirements, *Campylobacter* species are often isolated from a wide range of sources including animal digestive tracts, freshwater reservoirs and packaged food products (Horrocks et al., 2009; Zhao et al., 2015; Dearlove et al., 2016). While many *Campylobacter* strains are carried by host organisms asymptomatically, a subset of strains can cause gastric enteritis in humans (Lee and Newell, 2006; Pascoe et al., 2020). This *Campylobacter*-associated disease is known as campylobacteriosis and is the leading cause of gastric enteritis worldwide (World Health Organization, 2018). In the United States, a 2019 report identified *Campylobacter* species as the most common foodborne pathogen with an incidence of 19.5 infections per 100,000 people (Tack et al., 2019). While *Campylobacter* bacteria are found in a wide range of sources, human exposure to retail meats has been identified as a major risk factor for campylobacteriosis (Osimani et al., 2017).

The contribution to human campylobacteriosis is not spread evenly among *Campylobacter* species. The two most common *Campylobacter* species associated with foodborne illness are *C. jejuni* and *C. coli*. These species are the causative agents in > 85% and 5–10% of human campylobacteriosis, respectively (Patrick et al., 2018). Data from the National Antimicrobial Resistance Monitoring System (NARMS) shows that these species differ both in their antibiotic resistance profiles and their prevalence among food animal sources (Food and Drug Administration, 2021). Antibiotic resistance profiles of *C. coli* from retail meats are more commonly identified as resistant to at least one class of antibiotics, with resistance to macrolides, lincosamides, and ketolides showing a noticeable difference between species (Zhao et al., 2010; Food and Drug Administration, 2021). NARMS surveillance data of food animal cecal samples in the US reveals *C. coli* as the dominant *Campylobacter* species in swine and poultry samples (Dessai, 2017).

While *C. coli* isolation rates are comparable between cecal samples of swine and chicken, differences in post-slaughter processing practices can affect their isolation rate from retail meats of these animals. In communities not localized near food animals, exposure to retail meat products is a greater contributor to the burden of human campylobacteriosis than exposure to the food animals themselves (Rosner et al., 2017). Among the retail meats, retail poultry represents the greatest risk factor for human infection (Food and Drug Administration, 2010; Rosner et al., 2017). While isolates were readily recovered from the caeca of cattle and swine, *C. coli* were rarely isolated (<1% prevalence) from beef and pork retail meat products. As a result, screening for *Campylobacter* species in retail beef and pork products was discontinued in NARMS retail meat program in 2008. While the prevalence of *C. coli* is low among these food sources, the percentage of cattle and swine populations colonized with *C. coli* implicate them as potential reservoirs for *C. coli* infection in humans (Dessai, 2017; Plishka et al., 2020).

Retail meat products may be contaminated with *C. coli* originating from other sources through a variety of routes (Rasschaert et al., 2020). For example, studies have detailed how contact between infected animals during transport, airborne contamination in processing plants, bacterial contamination of contact surfaces at processing sites or even transfer of material from one sample to another during mechanical separation all may contribute to cross-contamination of poultry products (Battersby et al., 2016; Oliveira et al., 2020; Rasschaert et al., 2020; Konduru et al., 2021). With the advent of molecular surveillance methods, individual strains may be identified and tracked throughout the food processing chain. One method that is used for source tracking is core genome multilocus sequence typing (cgMLST) (Maiden et al., 2013). The core genome of *Campylobacter* species is defined by 1,343 loci as determined by Cody et al. (2017). Allele profiles of these 1,343 loci have sufficient diversity throughout the population of *Campylobacter* species that they can successfully be used for strain typing using a cgMLST schema (Hsu et al., 2020). Strains with similar allele profiles can be clustered into groups by allowing for a threshold of allelic differences between strains. Our previous study evaluating *C. jejuni* populations using core genome MLST with an allele difference of 200 (cgMLST_200_) showed an excellent correlation with traditional MLST typing and had a greater utility for source attribution and correlation with AMR profiles (Hsu et al., 2020). This method classifies strains into different groups if the allelic identity of more than 200 of the 1342 loci of the *Campylobacter* core genome differ between strains.

Recently, a minimal multilocus distance (MMD) analysis of *Campylobacter* genomes was demonstrated to be effective for source attribution (Pérez-Reche et al., 2020). This MMD method can be optimized to process the same 1,343 loci of the *Campylobacter* core genome as the cgMLST_200_ typing scheme to classify strains. Unlike the cgMLST_200_ scheme, though, the core genomes of individual strains are used to generate the strain clusters that define each source. This can be used for source attribution by comparing the core genomes from strains of an unknown source to the core genome model of each known isolation source and obtaining the probabilities of the strain having originated from each. While this method has the benefit of targeting associations between the core genome and defined metadata categories (e.g., isolation source), it is not currently optimized to account for distinct subpopulations within a strain cluster. That is to say, while the MMD method is well suited to identify if a strain originated from a chicken source, it may not be able to discriminate between the antibiotic-resistant subpopulations found within the chicken dataset. The objective of this study was to use both cgMLST and MMD methods to characterize the relative contributions of *C. coli* sources to act as reservoirs for antibiotic resistance genes and the relative contribution of each source to human campylobacteriosis.

## Materials and Methods

### Bacterial Strains, Sequencing, and AMR Identification

Whole genome sequencing data representing 3,432 *C. coli* isolates from environmental sources including water, soil and wild birds, food animal sources, human and retail meat sources were collected through the NARMS program, the National Center for Biotechnology Information (NCBI) database and the European Nucleotide Archive (ENA) database. Sequences of human (*n* = 285) and environmental source (*n* = 145) isolates were downloaded directly from their respective online databases. Food animal isolates were obtained through cecal sampling by the United States Department of Agriculture Food Safety and Inspection Service (USDA-FSIS) as part of NARMS animal arm and consisted of 964 cattle, 898 swine, 475 chicken and 296 turkey isolates of *C. coli*. Retail meat isolates collected through the NARMS retail meat program contained 374 chicken and 11 turkey isolates. All strains included in this study were isolated between 1999 and 2019. Genomic DNA was extracted using the Qiagen DNeasy Blood and Tissue kit (Qiagen, Gaithersburg, MD) and genomes were sequenced on an Illumina MiSeq using v2 or v3 chemistry (Illumina, San Diego, CA).

*C. coli* genomes were assembled using the CLC Genomics Workbench version 8.0 (CLC bio Aarhus, Denmark). Contigs less than 200 bp were removed prior to assembly and error correction was performed using the map reads back to contigs method. AMR determinants, including both resistance genes and point mutations, were identified with AMRFinder using database version 2020-09-30.1 (Feldgarden et al., 2019). Coverage and identity parameters were set to default values, using a minimum coverage of the reference protein of 0.5 and a minimum identity for BLAST hits of either the curated threshold value specific to the hit, or 0.9 if no curated threshold value was present.

### Core Genome MLST

The cgMLST groups for the *C. coli* dataset were assigned as previously described using an in house script (Hsu et al., 2020). Briefly, the cgMLST type for each strain was assigned using the allele sequences of the 1,343 loci that define the *Campylobacter* core genome. A BLAST search was performed to identify the alleles for each locus and up to 100 missing alleles was allowed for each genome. For strains where sequence data at the cgMLST loci was missing, the loci was assigned a value of “N.” Following cgMLST designation, the distance between core genomes was calculated based on the pairwise distance between allelic profiles and was measured in allelic difference. Strains were organized into groups allowing for a 200-allele difference between strains using a single-linkage clustering method.

The core genomes were used to generate a minimum spanning tree of the *C. coli* population using GrapeTree v1.5.0. A single table containing the core genome allele identities for each strain was used to generate the minimum spanning tree using GrapeTree v1.5.0. Each row of the table corresponded to a single strain and the table contained 1,343 columns, each representing one core genome loci. The cells were populated with the allele identity for each strain and loci pair. Using the MSTree V2 algorithm, the pairwise dissimilarity of core genome loci was compared between strains and used to generate the minimum spanning tree.

### Source Attribution

A source attribution model of human-pathogenic and retail meat *C. coli* isolates was generated using the MMD methodology developed by Pérez-Reche et al. (2020). Within this project, MMD source attribution was used to evaluate the dissimilarity of cgMLST loci between isolates of a known source, such as the population of *C. coli* isolated from chicken caeca, to those isolated from humans or retail meat samples. The pairwise dissimilarity, quantified as the Hamming distance, was compared between all known sources and the individual strains from human or retail meats to determine the likelihood of having originated from each of the sources.

The core genome multilocus sequence profiles and the known-source metadata of the 3,234 *C. coli* isolates were evaluated using the MMD R script. Source attribution was determined by modifying the MMD configuration file to include the isolation sources present in the isolate metadata file and setting the source attribution flag as the analysis to be performed. MMD self-attribution used the same dataset as source-attribution, with the configuration file modified to flag self-attribution as the analysis and a user-determined source as the target for self-attribution. Self-attribution analysis then generated cgMLST models for each isolation source and mapped he self-attribution target population back to the isolation source their cgMLST most closely resembles.

## Results

### Antibiotic Resistance Profiles of *C. coli*

**FIGURE 1:**
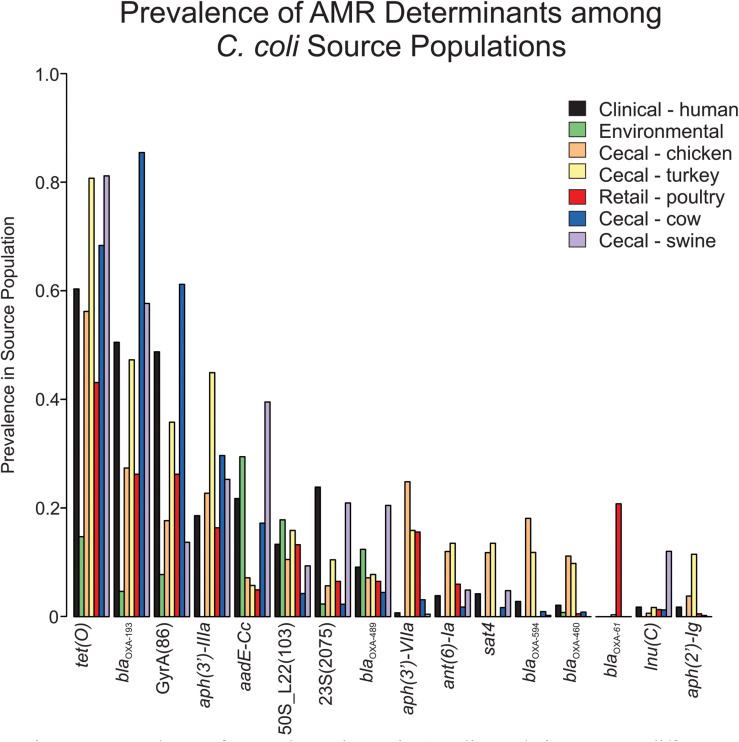
Prevalence of AMR determinants in *C. coli* populations among different isolation sources.

Screening *C. coli* sequence data with AMRFinder revealed 50 distinct alleles of antibiotic resistance genes and resistance-associated mutations among the isolates. While *tet(O)*, *bla*_*OXA*__–__193_ and *aph(3’)-IIIa* were the three most common antibiotic resistance genes, they were underrepresented in the environmental isolate subpopulation (Figure 1). The *bla*_*OXA*__–__61_ gene was almost exclusively found in retail poultry isolates with only a single instance recovered from a cecal turkey isolate. While *bla*_*OXA*__–__594_ and *bla*_*OXA*__–__460_ were most common among cecal chicken and turkey populations at a rate of 9.8–18.1%, *bla*_*OXA*__–__594_ was missing from the retail poultry isolates and bla_*OXA*__–__460_ was only present in 0.5% of the population. A set of 344 *bla*_*OXA*_ alleles could not be annotated at the allele level due to incomplete sequence and were not included in this project. Ribosomal mutations resulting in the substitutions L23_A2075G and L22_A103V associated with macrolide and lincosamide resistance did not show exclusivity for any isolation source, though L23_A2075G was more strongly represented in swine and human isolates (Supplementary Table 1). L23_A2075G was present at the greatest rate in cecal swine isolates at 20.9% and L22_A103V was most prevalent in environmental sources at 17.8%. The GyrA_T86I substitution associated with resistance to fluoroquinolones was more common, present in cecal cattle (61.1%), human (48.8%), cecal turkey (35.8%), retail poultry (26.2%), cecal chicken (17.7%), and environmental (7.8%) isolates.

### Core Genome Multilocus Sequence Typing of *C. coli*

**FIGURE 2:**
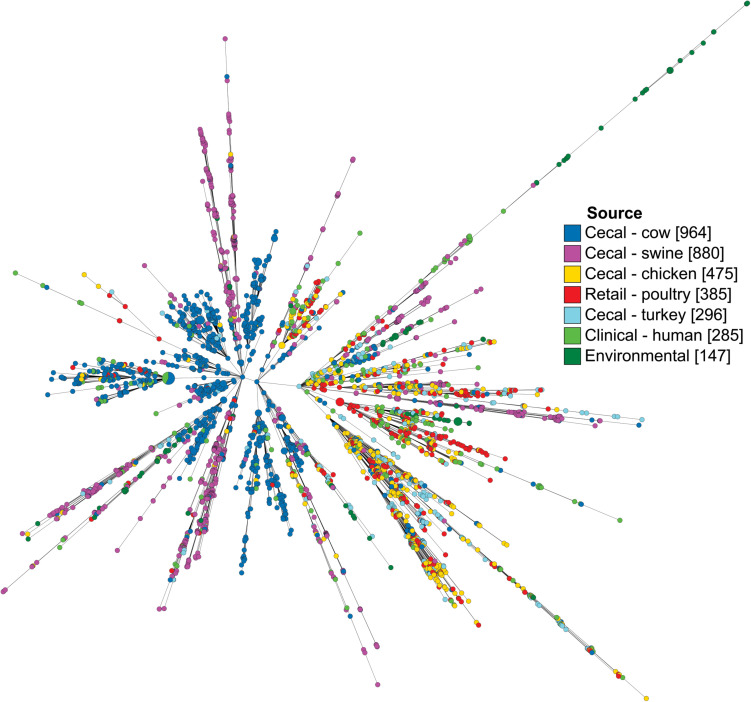
Minimum spanning tree of *C. coli* dataset as determined by their core genome. Each circle represents a cgMLST group and the size of the circle is proportional to the number of isolates in that group. Tree was generated in GrapeTree using a log depiction of branch length.

*Campylobacter coli* isolates were characterized by their core genome of 1,343 loci and visualized as a minimum spanning tree (Figure 2). Isolates from the same source co-localized among branches, though few branches were exclusive to a single source. Notably, while isolates from human sources were observed throughout the tree, they were present at the highest concentrations on the same branch that favored poultry isolates, including both cecal and retail isolates.

### cgMLST_200_ Typing of *C. coli*

Organizing *Campylobacter* strains by core genome into groups that allow a 200-allele difference among isolates is an effective subtyping method for differentiating strains both among and between *Campylobacter* species. Our cgMLST analysis generated 1,085 cgMLST_200_ groups from our 3,432 *C. coli* isolates. Within the 1,085 cgMLST_200_ groups, 818 contain only one isolate each and were designated as singletons. The majority of these singleton strains were isolated from the caeca swine, indicating greater sequence diversity in the swine population compared to other isolation sources. Non-singleton cgMLST_200_ group analysis revealed that 126 of the remaining 267 cgMLST_200_ groups were identified among multiple sources. These results show that 88.4% of the cgMLST_200_ groups were specific to a single isolation source and only 11.6% could be found in multiple hosts.

**FIGURE 3:**
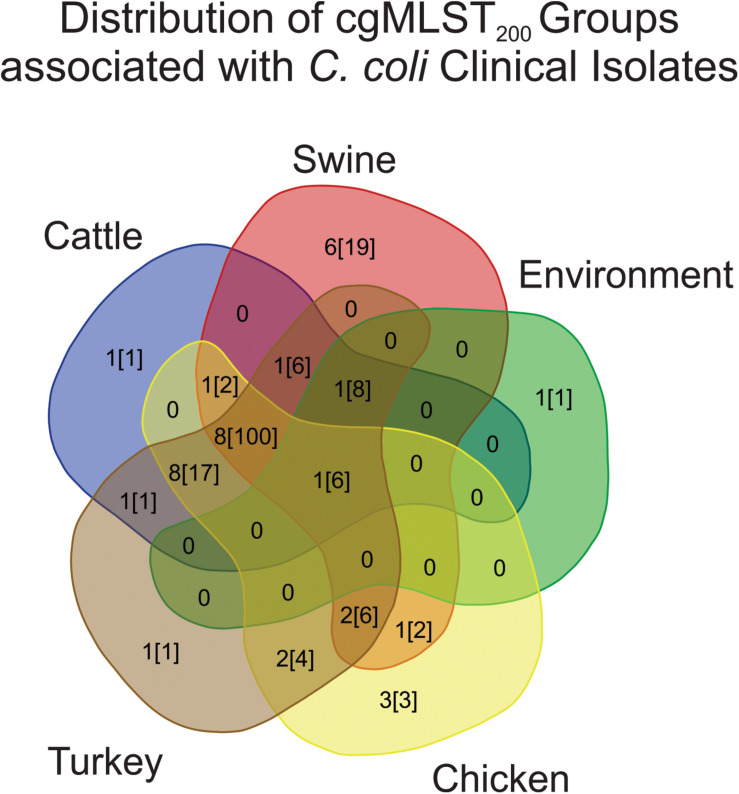
Distribution of cgMLST_200_ groups that contain human pathogenic strains of *C. coli*. The majority of human isolates belong to cgMLST_200_ groups containing isolates from a variety of sources. The first number listed in each source shows how many cgMLST_200_ groups contain human-pathogenic *C. coli*. The second number, in brackets, is the number of human-pathogenic strains in the cgMLST_200_ groups.

*Campylobacter* cgMLST_200_ classification was then used to evaluate the similarity of strains from humans to strains recovered from other sources. Evaluating the cgMLST_200_ groups from each source showed that 25/285 *C. coli* clinical isolates belonged to a cgMLST_200_ group associated with only one other source (Figure 3). The largest contributor to human-pathogenic strains was a set of 8 cgMLST_200_ groups that contained isolates from cattle, chicken, turkey and swine sources. These accounted for 100/285 *C. coli* strains isolated from humans.

Strains recovered from swine were least similar to human isolates, with only 7.7% of the population belonging to human-pathogenic cgMLST_200_ groups (Table 1). Evaluating turkey, chicken and environmental populations revealed that 33.6, 26.3, and 20.1% of their populations shared cgMLST200 groups with humans, respectively. Cattle isolates showed the strongest representation of human-pathogenic cMLST_200_ groups, with 87.7% of the strains belonging to one of these groups. A single human-pathogenic cgMLST_200_ group (cgMLST_200_-234) also contained ∼two-thirds of the entire cattle population and accounted for the large difference in proportion between cattle and other sources (Supplementary Table 2).

**TABLE 1:** Isolation source composition of cgMLST200 groups containing clinical strains of *C. coli*.

Source	# of isolates	Total # of cgMLST_200_ groups	cgMLST_200_ groups shared with Human	Source population within human-pathogenic cgMLST_200_ groups
Swine	898	617	21	69 (7.7%)
Environment	129	83	3	26 (20.1%)
Cattle	964	117	22	845 (87.7%)
Chicken	849	233	26	223 (26.3%)
Turkey	307	123	25	103 (33.6%)

Several cgMLST_200_ groups showed strong correlation with the presence of antibiotic resistance determinants (Supplementary Table 1). Seven antibiotic resistance genes were conserved at > 95% prevalence in 6 cgMLST_200_ groups (Table 2). Further, strains from these cgMLST_200_ groups retained the corresponding antibiotic resistance genes regardless of the isolation source. Within the cgMLST_200_-5 group, *bla*_*OXA*__–__489_ was found in strains isolated from swine, cattle, turkey, human and environmental sources. The cgMLST_200_-5 group was notable as well because 50/50 strains contained a mutation in 50S_L22A103V that can confer resistance to macrolide antibiotics. In addition, all 16 isolates in cgMLST_200_-266 group had a mutation in GyrAT86I associated with resistance to fluoroquinolone antibiotics (Table 3).

**TABLE 2:** Prevalence of AMR genes in cgMLST_200_ groups.

cgMLST_200_ group	AMR gene	Prevalence	Isolation source	cgMLST_200_ group strains from isolation source with AMR gene
cgMLST_200_-435	*aph*(3)*-VIIa*	17/17 (100%)	Retail poultry	17/17
cgMLST_200_-654	*aph*(3)*-VIIa*	89/92 (96.7%)	Cecal chicken	51/54
			Cecal cow	6/6
			Cecal swine	1/1
			Cecal turkey	31/31
cgMLST_200_-266	*aph*(2)*-If*	16/16 (100%)	Cecal chicken	1/1
			Cecal turkey	15/15
cgMLST_200_-5	*bla*_*OXA*__–__489_	49/50 (98%)	Cecal cow	18/18
			Cecal swine	1/1
			Cecal turkey	9/9
			Human	7/8
			Environmental	14/14
cgMLST_200_-597	*bla*_*OXA*__–__460_	37/38 (97.4%)	Cecal chicken	22/22
			Cecal cow	1/2
			Cecal turkey	9/9
			Human	5/5
cgMLST_200_-461	*ant*(6)*-Ia*	23/23 (100%)	Cecal chicken	6/6
			Cecal cow	4/4
			Cecal turkey	13/13
cgMLST_200_-461	*sat4*	22/23 (95.7%)	Cecal chicken	6/6
			Cecal cow	3/4
			Cecal turkey	13/13

**TABLE 3:** Prevalence of AMR mutations in cgMLST_200_ groups.

cgMLST_200_ group	AMR substitution	Prevalence	Isolation source	cgMLST_200_ group strains from isolation source with AMR substitution
cgMLST_200_-266	GyrAT86I	16/16 (100%)	Cecal turkey	15/15
			Cecal chicken	1/1
cgMLST_200_-84	GyrAT86I	14/15 (93.3%)	Retail poultry	14/14
			Environmental	0/1
cgMLST_200_-248	GyrAT86I	146/184 (79.3%)	Cecal chicken	2/6
			Cecal cow	117/143
			Cecal swine	1/2
			Cecal turkey	2/3
			Human	24/30
cgMLST_200_-221	GyrAT86I	49/65 (75.4%)	Cecal chicken	24/28
			Cecal cow	5/7
			Cecal swine	2/2
			Cecal turkey	6/12
			Human	12/16
cgMLST_200_-234	GyrAT86I	452/676 (66.9%)	Cecal chicken	4/8
			Cecal cow	405/637
			Cecal swine	2/7
			Cecal turkey	6/10
			Human	6/14
cgMLST_200_-5	L22A103V	50/50 (100%)	Cecal cow	18/18
			Cecal swine	1/1
			Cecal turkey	9/9
			Human	8/8
			Environmental	14/14
cgMLST_200_-558	L22A103V	23/24 (95.8%)	Cecal chicken	14/15
			Cecal cow	5/5
			Cecal swine	1/1
			Cecal turkey	2/2
			Human	1/1
cgMLST_200_-597	L22A103V	30/38 (78.9%)	Cecal chicken	16/22
			Cecal cow	2/2
			Cecal turkey	9/9
			Human	3/5
cgMLST_200_-266	L23A2075G	16/16 (100%)	Cecal turkey	15/15
			Cecal chicken	1/1

### Core Genome Minimal Multilocus Distance Analysis

MMD methodology was used to determine the contribution of each *Campylobacter* source to human campylobacteriosis. This approach differs from cgMLST_200_ typing in that MMD generates a model of core genome profiles that best represent each isolation source before performing source attribution of the *C. coli* clinical isolates. Because of this difference in methodology, source attribution calls of MMD can differ from those determined by cgMLST_200_ typing. A population analysis of human-pathogenic *C. coli* using MMD highlights cecal chicken isolates as the greatest contributor to campylobacteriosis and indicates cecal swine sources as the weakest contributor (Table 4). Further population analysis revealed that isolates collected from swine and environmental sources showed highest likelihood of correct self-attribution during method validation (Supplementary Figure 1).

**TABLE 4:** Source attribution of human-pathogenic *C. coli* population.

	Likelihood of attribution	2.5 percentile	97.5 percentile
Cattle—cecal	0.198 ± 0.02	0.16	0.238
Environmental	0.209 ± 0.021	0.17	0.25
Chicken—cecal	0.311 ± 0.016	0.28	0.344
Turkey—cecal	0.185 ± 0.011	0.163	0.206
Swine—cecal	0.098 ± 0.012	0.075	0.122

**FIGURE 4:**
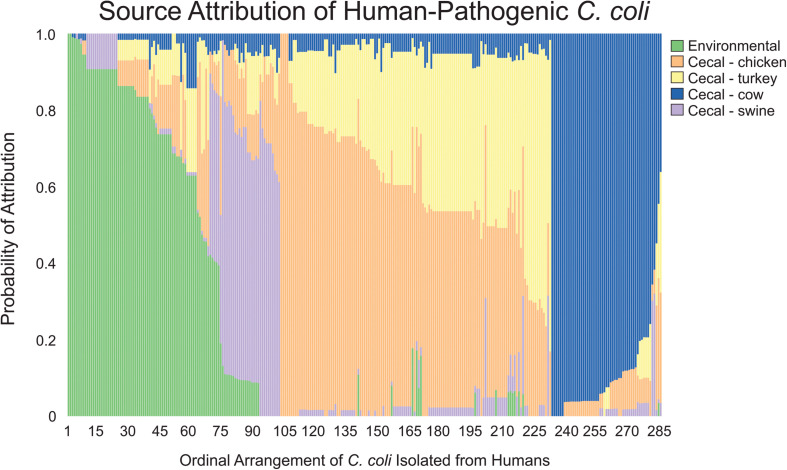
Source attribution of human-pathogenic *C. coli* to environmental and food animal cecal sources. Each bar represents a single *C. coli* isolate obtained from a human source and the color composition of the bar shows the likelihood of the strain as originating from the evaluated sources.

In contrast to the population-wide analysis noted above, we then employed MMD to perform source attribution for the individual strains that were isolated from humans (Figure 4). *Campylobacter* contamination of retail meats can occur at multiple points from slaughter processing to packaging for retail consumption (Oliveira et al., 2020). To address the potential of a heterogeneous source population obtained from retail meats, clinical isolates were only sourced to cecal isolate populations that were obtained prior to processing. Only 4.2% of the clinical isolates could be attributed to a single source with 100% likelihood and 11.9% of the population could be attributed at >95% likelihood. *C. coli* were attributed to chicken and turkey sources at 36.5 and 9.1%, respectively, for a combined total of 45.6% from poultry sources. A total of 23.9% of human isolates were attributed to environmental sources and the remaining 18.6 and 11.9% were attributed to cattle and swine, respectively.

**FIGURE 5:**
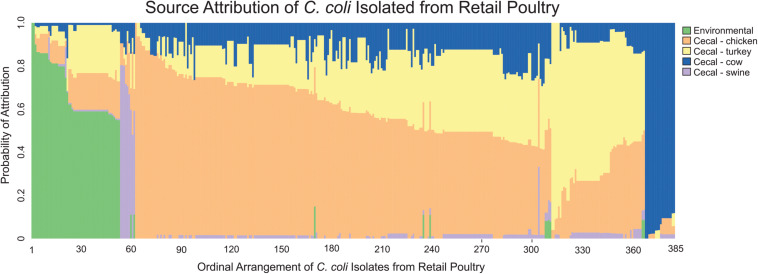
Source attribution of *C. coli* isolated from retail meats to environmental and food animal cecal sources. Each bar represents a single *C. coli* isolate obtained from a retail meat source and the color composition of the bar shows the likelihood of the strain as originating from the evaluated sources.

MMD was then used to determine the likely source of *C. coli* isolates from retail poultry samples. As expected, analysis of the individual strains revealed that 79.2% of *C. coli* recovered from retail poultry meats showed the strongest likelihood of attribution to cecal chicken or cecal turkey isolates (Figure 5). Interestingly, environmental sources showed the next highest likelihood of attribution, accounting for 13.8% of the population while the remaining strains were attributed to cattle and swine at 4.7 and 2.3%, respectively.

**FIGURE 6:**
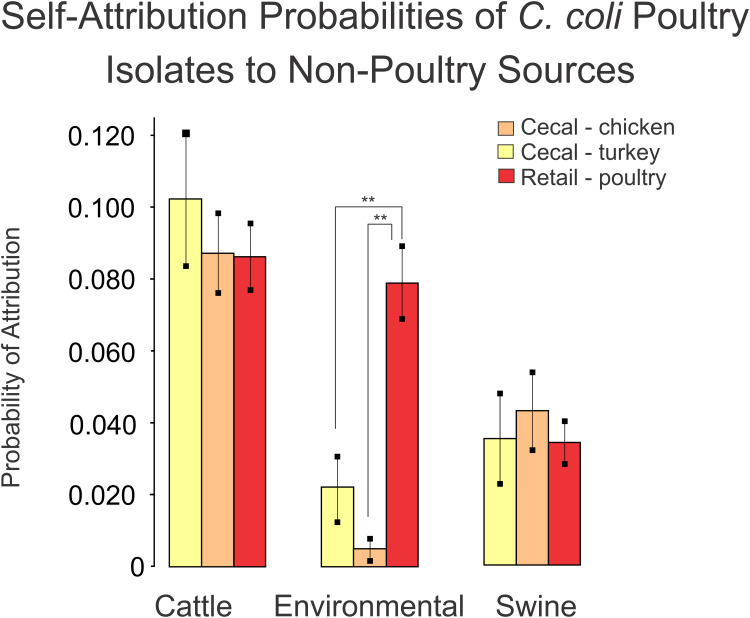
A comparison of self-attribution results for poultry datasets to non-poultry sources as an indicator of population similarity. Blinded validation of poultry datasets misattributed poultry isolates to cattle and swine sources at similar rates. Misattribution of retail poultry isolates to environmental sources occurred at a higher rate (**p < 0.001) than the misattribution of cecal chicken or cecal turkey isolates.

As a final step, we compared the self-attribution results from the turkey, chicken and retail poultry isolates between the non-poultry sources using the Tukey multiple comparison of means. This comparison was made to determine if any of the poultry sources were preferentially identified as having originated a non-poultry source to indicate a source of contamination. Turkey, chicken and retail poultry isolates were attributed to a cattle source at a similar rate of 8.7–10.3%. Similarly, turkey, chicken and retail poultry isolate were attributed to a swine source at a range of rates from 3.4 to 4.3%. Attribution to environmental sources showed a difference in attribution rates between turkey, chicken and retail poultry isolates. Chicken isolates were attributed to environmental sources at a rate of 0.4% and turkey isolates at 2.2% (Figure 6). Retail poultry isolates, however, were attributed to environmental sources at a rate of 8.0%.

## Discussion

In this study, we used the *C. coli* core genome to categorize isolates with two independent methods. The first method, cgMLST_200_, is a subtyping scheme that organizes strains based on the similarity of the core genomes. We have demonstrated how strains with similar core genome profiles can show strong association with specific metadata categories, such as AMR determinants or isolation host. The second method, MMD analysis, evaluates the core genomes of all strains of a given metadata group (e.g., source) and establishes core genome profiles of each group. Using this method, we were able to evaluate each strain from our human and retail meat populations and identify the most likely source for attribution.

Comparing the results from each method reveals the methods’ relative strengths and limitations. The metadata-agnostic cgMLST_200_ typing schema clusters related strains into groups with 100% probability of membership, but the groups themselves are not necessarily congruent with any metadata feature. This makes cgMLST_200_ analyses well suited for our evaluation of populations against large metadata sets, such as associations to the entire library of AMR determinants. Another benefit of the cgMLST_200_ typing schema is that it provides a common naming system that categorizes *Campylobacter* strains by core genome similarity and can be applied to strains where only sequence data is known. The MMD approach uses metadata to define source attribution groups and provides the relative likelihood of any sample originating from any of the attribution groups. While this method is optimized to identify the likely source of a *C. coli* strain, it requires that the sources be made up of distinct populations. This trait prevented us from attributing human-pathogenic *C. coli* to the retail poultry isolates, which were a composite of chicken, turkey and putative contaminants from other sources.

The presence of strains from the retail poultry population attributed to swine, environmental and cattle isolates implicate retail poultry consumption as one route by which humans may be exposed to *C. coli* from environmental contaminants. A comparison of self-attribution results among retail poultry isolates to cecal samples showed no difference in their attribution rates to swine and cattle. This indicates that fraction of poultry isolates attributed to swine and cattle is not greatly affected by events post-slaughter. The fraction of *C. coli* isolated from all poultry samples attributed to environmental sources reveals that strains recovered from retail poultry are more likely than cecal samples to be attributed to our dataset of *C. coli* generated from environmental sources. Because there is variation in the rate of attribution to only one of the three sources, this indicates that the discrepancy is not due to allelic variability within the retail poultry isolates alone.

Comparing the mean cgMLST_200_ group size was one indicator of genomic diversity among our attribution sources. Mean cgMLST_200_ group size increased as core genome diversity between strains decreased below the 200-allele difference threshold. One limitation of evaluating genomic diversity using this metric is that interpretation of the results is dependent on the number of allele differences allowed. This limitation is illustrated when comparing our results between cattle and swine isolates. Although swine and cattle sources contained a similar number of isolates, there were ∼5 times as many cgMLST_200_ groups in swine. A closer look at the cattle data revealed that 80% of the cattle strains were classified into only two cgMLST_200_ groups. Taking this unequal distribution of strains among cgMLST_200_ groups into account, the genomic diversity of *C. coli* isolated from cattle outside these two cgMLST_200_ groups is similar to the swine isolates. While the majority of cattle isolates were classified into similar cgMLST_200_ groups, the opposite was observed in the swine isolates. Of the 617 cgMLST_200_ groups present in swine, 505 groups contained only 1 isolate each. Evaluating these data between isolation sources using a cgMLST_200_ schema alone provides a general metric for comparing the genomic diversity among isolates from different food sources.

While the MMD method provided detailed predictions of source attribution, the cgMLST_200_ typing scheme was able to cluster several large groups of strains with conserved AMR determinants. Combining these two methods to identify subpopulations encoding antibiotic resistant determinants was relevant to this project as, with the exception of *bla*_*OXA*__–__61_ in retail poultry, the resistance determinants did not show a bias for any isolation source. This is in contrast with earlier AMR profiling of *C. jejuni* from food animals. The *C. jejuni* study showed that chromosomal mutations associated with macrolide resistance were absent from the cattle population while here we show these chromosomal mutations are present in *C. coli* from all isolation sources. This point is noteworthy because *C. coli* and *C. jejuni* are often isolated from the same environment and experience similar sets of evolutionary selective pressures.

In this study, we have shown how cgMLST_200_ typing can be used to inform the likelihood of certain antimicrobial resistance determinants in subpopulations of *C. coli*. Further, both methods used for source attribution indicated that swine sources contributed least to the human burden of campylobacteriosis. This is despite a high prevalence of *C. coli* isolated from swine cecal samples. The core genomes of poultry isolates showed the highest similarity to human isolates while environmental, cattle and swine isolates were increasingly dissimilar. Without supporting epidemiological data, however, we are unable to claim that any of the *C. coli* isolated from humans originated from a particular source. Data generated from these methods allow us to evaluate the genomic similarity of human-pathogenic *C. coli* strains to each of the isolation sources. Using both cgMLST_200_ and MMD analyses, we have demonstrated the utility of large-scale genomic analysis of *C. coli* for identifying risk factors of campylobacteriosis, including identifying the likely source of antimicrobial resistant *C. coli* infections.

## Data Availability Statement

The datasets presented in this study can be found in online repositories. The names of the repository/repositories and accession number(s) can be found in the article/Supplementary Material.

## Author Contributions

ES, LH, and SZ were involved in the conception of the project and study design. CM, JH, SM, and SY collected sequence and metadata. SM was responsible for database organization and curation. C-HH, LH, SM, and SZ performed data analysis. C-HH, CM, ES, GT, JH, LH, QZ, SM, SY, and SZ revised and reviewed the manuscript. All authors contributed to the article and approved the submitted version.

## Disclaimer

The views expressed in this article are those of the authors and do not necessarily reflect the official policy of the Department of Health and Human Services, the U.S. Food and Drug Administration, or the U.S. Government. Reference to any commercial materials, equipment, or process does not in any way constitute approval, endorsement, or recommendation by the Food and Drug Administration.

## Conflict of Interest

The authors declare that the research was conducted in the absence of any commercial or financial relationships that could be construed as a potential conflict of interest.
