# Supplementary material for: Core Genome MLST for Source Attribution of Campylobacter coli
Source: Front Microbiol. 2021 Jul 13;12:703890. doi: 10.3389/fmicb.2021.703890 (PMC8313984; doi:10.3389/fmicb.2021.703890)
Supplement: Supplementary file 4 [file Image_1.PDF]

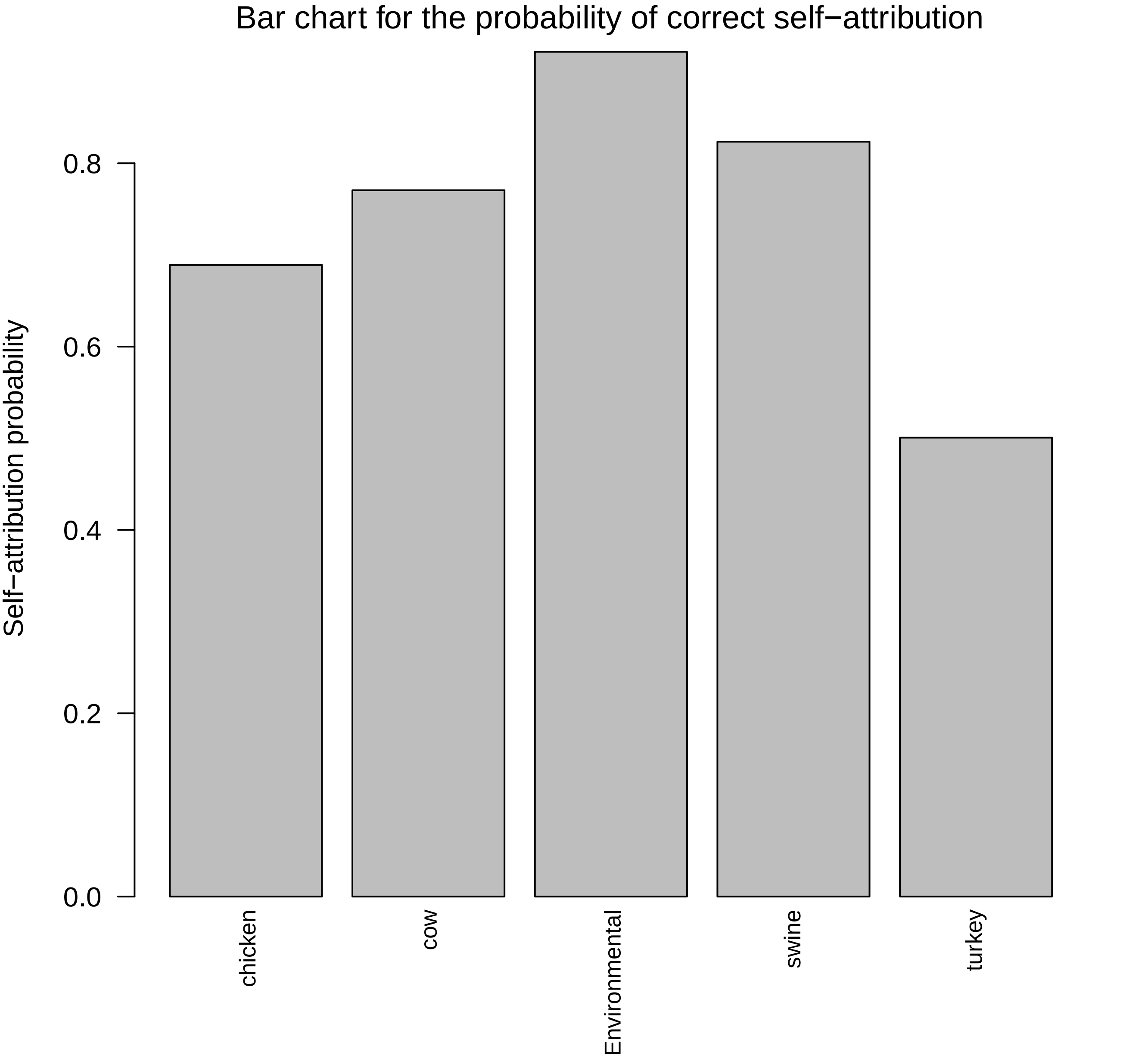

Figure S1. This bar chart represents the probability that an isolate from a known source will be correctly attributed to that source during the validation phase of MMD source attribution
